# Self-compassion mediates the relationship between self-esteem and social anxiety symptoms in socially anxious individuals

**DOI:** 10.1192/j.eurpsy.2021.1639

**Published:** 2021-08-13

**Authors:** P. Holas, M. Kowalczyk, I. Krejtz, K. Wisiecka, T. Jankowski

**Affiliations:** 1 Faculty Of Psychology, University of Warsaw, Warsaw, Poland; 2 Department Of Psychology, University of Warsaw, Warsaw, Poland; 3 Psychology, SWPS University of Social Sciences and Humanities, Warsaw, Poland; 4 Psychology, John Paul II Catholic University of Lublin, Lublin, Poland

**Keywords:** social anxiety, Self-compassion, SAD, self-esteem

## Abstract

**Introduction:**

Fear of evaluation and a negative view of the self are the key characteristics of social anxiety, which is one the most prevalent anxiety problem. Self-esteem refers to views of oneself, including individual’s personal feelings towards self, whereas self-compassion refers to caring attitude toward oneself. Both constructs are two distinct positive views of the self, and were found to be related to each other, well-being and good mental health. To date, however, little is known, how they interplay in people with predominantly negative view of themselves, that is in socially anxious individuals.

**Objectives:**

The current research aims at evaluating how social anxiety interacts with self-esteem and self-compassion and to assess whether self-compassion, mediates the relationship between social anxiety and self-esteem.

**Methods:**

In this research, 388 adult participants with elevated social anxiety level (LSAS score M = 81.47, SD = 21.20) were recruited via open calls posted on the Internet and completed measures of social anxiety, self-compassion, and self-esteem.

**Results:**

In accordance with the view that individuals with social anxiety tend to have negative mental representation of the self, we found that both self-esteem and self-compassion correlated negatively with social anxiety, and positively with one another. More importantly, self-compassion partially mediates the relationship between self-esteem and social anxiety.

**Conclusions:**

These findings suggest that self-compassion may play an important role in buffering against social anxiety and suggest that enhancing self-compassion might be beneficial for reducing symptoms of social anxiety

**Disclosure:**

No significant relationships.

